# Evolutionary and synteny analysis of *HIS1*, *BADH2*, *GBSS1*, and *GBSS2* in rice: insights for effective introgression breeding strategies

**DOI:** 10.1038/s41598-024-55581-w

**Published:** 2024-03-04

**Authors:** Insu Lim, Yong-Jin Park, Jungmin Ha

**Affiliations:** 1 Department of Plant Science, Gangneung-Wonju National University, Gangneung, South Korea; 2 Department of Plant Sciences, Kongju National University, Yesan, 340-702 Korea

**Keywords:** Plant breeding, Plant evolution, Plant genetics

## Abstract

The key genes *BADH2*, *GBSS1, GBSS2*, and *HIS1* regulate the fragrance, starch synthesis, and herbicide resistance in rice. Although the molecular functions of four genes have been investigated in the *Oryza sativa* species, little is known regarding their evolutionary history in the *Oryza* genus. Here, we studied the evolution of four focal genes in 10 *Oryza* species using phylogenetic and syntenic approaches. The *HIS1* family underwent several times of tandem duplication events in the *Oryza* species, resulting in copy number variation ranging from 2 to 7. At most one copy of *BADH2*, *GBSS1*, and *GBSS2* orthologs were identified in each *Oryza* species, and gene loss events of *BADH2* and *GBSS2* were identified in three *Oryza* species. Gene transfer analysis proposed that the functional roles of *GBSS1* and *GBSS2* were developed in the Asian and African regions, respectively, and most allelic variations of *BADH2* in *japonica* rice emerged after the divergence between the Asian and African rice groups. These results provide clues to determine the origin and evolution of the key genes in rice breeding as well as valuable information for molecular breeders and scientists to develop efficient strategies to simultaneously improve grain quality and yield potential in rice.

## Introduction

Rice is a staple food crop for half of the world population, contributing to nearly 20% of the total calorie intake of humans^1^. As the global population is predicted to reach almost 10 billion by 2050^2^, the development of novel high-yield and superior-quality rice cultivars is urgently required to meet the future global food demand^3^.

Rice is one of the most extensively studied crop species because of its social and economic importance as well as its environmental impact^4^. Many genetic factors related to grain production such as starch biosynthesis and abiotic stress resistance, have captured the interest of researchers^5^, including the genes betaine aldehyde dehydrogenase (*BADH2,* Os08g0424500), granule-bound starch synthase 1 (*GBSS1,* Os06g0133000), granule-bound starch synthase 2 (*GBSS2,* Os07g0412100), and HPPD (4-hydroxyphenylpyruvate dioxygenase) inhibitor sensitive 1 (*HIS1,* Os02g0280700). *BADH2* encodes for the enzyme that biosynthesizes 2-acetyl-1-pyrroline, which is a potent rice flavor compound^6^. Fragrant type of rice cultivars have been identified to be determined by allelic variations of the *BADH2* gene^6^. *GBSS1* and *GBSS2* are responsible for the amylose content of rice by converting ADP-glucose into amylose instead of amylopectin in the starch biosynthesis pathway^7^. The amylose to amylopectin ratio is the most important physiological indicator of rice grain quality, particularly regarding the cooking and eating qualities^8,9^. *HIS1* imparts resistance to bTH benzobicyclon and other b-Triketone herbicides that are widely applied to weed control in rice paddy fields^10^. Because weed control of large-scale farming is highly dependent on herbicides, the wide-spectrum tolerance to multiple herbicides are essential traits for increased rice production with reduced labor.

*Oryza sativa* and *O. glaberrima* were independently domesticated in Asia and Africa, respectively, from different wild ancestors^1^. In general, wild species obtain various alleles for resistance or tolerance to environmental stresses as adaption to a broad biogeographical diversity^11,12^. This rich diversity of wild relatives allows for introgression breeding to be a prominent approach in rice to enhance agricultural traits such as resistance to biotic and abiotic stresses^13–15^. Several previous studies have reported that introgression of alleles from wild germplasms enhanced the productivity and grain quality in soybeans and tomatoes^16,17^. However, in rice, most introgression breeding strategies have focused on conferring pest and disease resistance to *O. sativa* species^13–15^.

The genus *Oryza* comprises of two cultivated species and 22 wild species with 11 representative genome types: six diploids (AA, BB, CC, EE, FF, and GG) and five polyploids (BBCC, CCDD, HHJJ, HHKK, and KKLL)^18^. *Oryza sativa* and *Oryza glaberrima*, cultivated species, belong to the AA group with six wild species (*Oryza nivara*, *Oryza rufipogon*, *Oryza barthii*, *Oryza glumaepatula*, *Oryza longistaminata*, and *Oryza meridionalis*). Wild *Oryza* species have been considered as potential genetic resources that carry valuable alleles which are not present in the cultivated species^19^. Regarding abiotic and biotic stress resistance, many rice breeders have characterized favorable alleles in wild *Oryza* species^11,12,20^, and numerous alleles conferring stress resistance have been introduced from wild *Oryza* species into elite cultivars^18,21^, as demonstrated by the chromosomal introgression of iron resistance from *O. meridionalis* into *O. sativa*^22^. These results propose that the stability and productivity of elite rice cultivars could be improved by gene transfer from wild germplasms. However, the genes related to grain quality and herbicide resistance in rice, such as *BADH2*, *GBSS1*, *GBSS2*, and *HIS1*, have only been investigated within the *O. sativa* group.

This study aimed to investigate the evolution and divergence of the *BADH2*, *GBSS1*, *GBSS2*, and *HIS1* gene families within the *Oryza* genus, including both cultivated and wild species. To provide a robust foundation for the evolution analysis of gene families, a phylogenetic tree was constructed among 10 *Oryza* species and macrosynteny was analyzed in four Asian *Oryza* species, including *O. sativa* ssp. *japonica* and *indica*, *O. nivara*, and *O. rufipogon*. Through a comprehensive phylogenetic and syntenic network approach, the evolutionary events and selection pressures were explored. The findings in this study contribute to a better understanding of the evolution of these target gene families and provide valuable insights for breeders in the selection of beneficial germplasm to develop more adaptive and productive rice cultivars.

## Results

### Phylogenetic analysis

**Figure 1:**
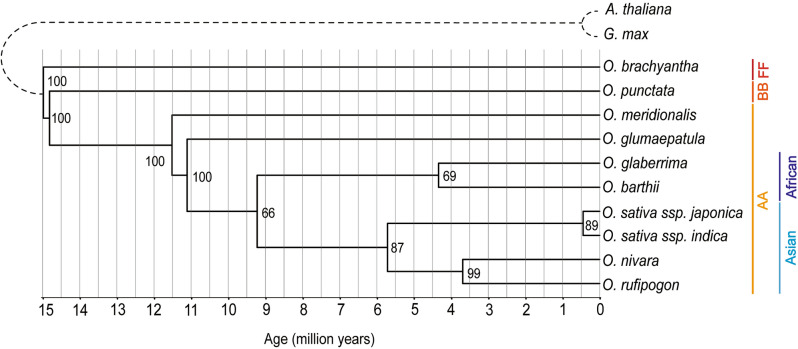
Phylogeny of 10 *Oryza* species and two dicot plants. The phylogenetic tree was constructed using the amino acid sequences of 50 true orthologs among 10 *Oryza* species and two dicot species as outgroups. The number of each node is the bootstrap value between nodes which is obtained from 1000 bootstrap replications. Divergence times within the genus *Oryza* were estimated by assuming 15 million years ago for the divergence point between *O. brachyantha* and the other *Oryza* species. *Mya* million years ago.

We constructed two phylogenetic trees using peptide and nucleotide sequences, respectively, using 50 true orthologs from 10 *Oryza* species with *A. thaliana* and *G. max* as outgroups (Fig. 1). There were no significant differences in topology between two phylogenetic trees (Fig. 1 and Fig. S2). The eight AA-genome *Oryza* species were clustered together, thereby separating from *O. punctata* (BB-genome) and *O. brachyantha* (FF-genome) (Fig. 1). Within the AA-genome clade, the African and Asian species were grouped separately and the Asian species were further separated into two groups, namely the cultivated (*O. sativa* ssp. *japonica* and *O. sativa* ssp. *indica*) and wild groups (*O. rufipogon* and *O. nivara*) (Fig. 1). Using the divergence time between *O. brachyantha* and the other *Oryza* species at 15 million years ago (mya) as the calibration point^1^, the divergence times for the branching points was calculated in the phylogenetic tree (Fig. 1). The estimated divergence time between the AA- and BB-genomes was 14.8 mya, 9.2 mya between the African and Asian clades, 3.6 mya between the Asian wilds, and 0.4 mya between the Asian cultivars (Fig. 1).

### Synteny analysis

**Figure 2:**
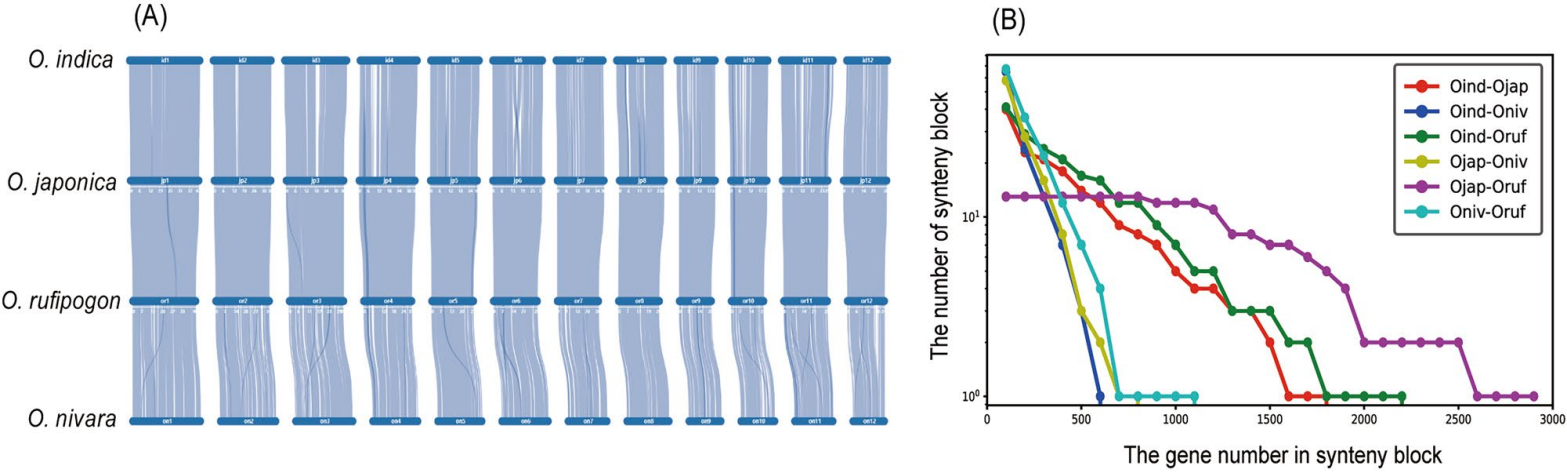
Synteny analysis between Asian rice groups. (**A**) Macrosynteny visualization of four Asian species ordered by *O. indica*, *O. japonica*, *O. rufipogon*, and *O. nivara*. (**B**) The distribution of the synteny block size of six species pairs show the degree of syntenic conservation. The x-axis indicates the number of genes required to call a synteny block and the y-axis indicates the number of synteny blocks between each species pair.

To further understand the evolutionary process during the domestication of cultivated rice, *O. sativa* ssp. *indica* and *O. sativa *ssp.* japonica*, we further investigated the macrosynteny among the four Asian species at the chromosomal-level (Fig. 2A). *O. sativa* species had a more conserved synteny with *O. rufipogon* than *O. nivara* (Fig. 2B). *O. sativa *ssp.* japonica* and *O. rufipogon* showed the most conserved synteny among the four species and its breakpoint was identified only once on chromosome 3 (Fig. 2). The conservation of synteny between *O. sativa* ssp. *indica* and *O. sativa *ssp.* japonica* was slightly lower than that of synteny between *O. sativa* ssp. *indica* and *O. rufipogon* (Fig. 2B).

### Identification of the *BADH2*, *GBSS1*, *GBSS2*, and *HIS1* gene families

Orthologous genes of *BADH2*, *GBSS1*, *GBSS2*, and *HIS1* were identified in the 10 *Oryza* species using clustering analysis based on the annotation information of the *O. sativa *ssp.* japonica* reference genomic sequence^23^. A total of 8, 10, 8, and 43 orthologous genes were identified as *BADH2*, *GBSS1*, *GBSS2*, and *HIS1* gene family members, respectively (Table 1). For *BADH2*, *GBSS1*, and *GBSS2*, in general, single-copy genes remained across the *Oryza* species (Table 1). *BADH2* was lost in *O. meridionalis* and *O. punctata* and *GBSS2* was lost in *O. brachyantha* and *O. meridionalis* (Table 1). While *GBSS1* was uniformly identified in every *Oryza* species without any loss events (Table 1). The *HIS1* family showed high variation in their copy number ranging from two (*O. brachatia* and *O. glumaetuala*) to seven (*O. sativa *ssp.* japonica* and *O. puctata*) (Table 1).

**Table 1:** Number of orthologs (*HIS1*, *BADH2*, *GBSS2*, and *GBSS2*) in the 10 *Oryza* species.

	*O. bar*	*O. bra*	*O. gla*	*O. glu*	*O. ind*	*O. jap*	*O. mer*	*O. niv*	*O. pun*	*O. ruf*
*HIS1*	4	2	6	2	3	7	4	5	7	3
*GBSS1*	1	1	1	1	1	1	1	1	1	1
*GBSS2*	1	0	1	1	1	1	0	1	1	1
*BADH2*	1	1	1	1	1	1	0	1	0	1

### Gene synteny analysis of *HIS1*

**Figure 3:**
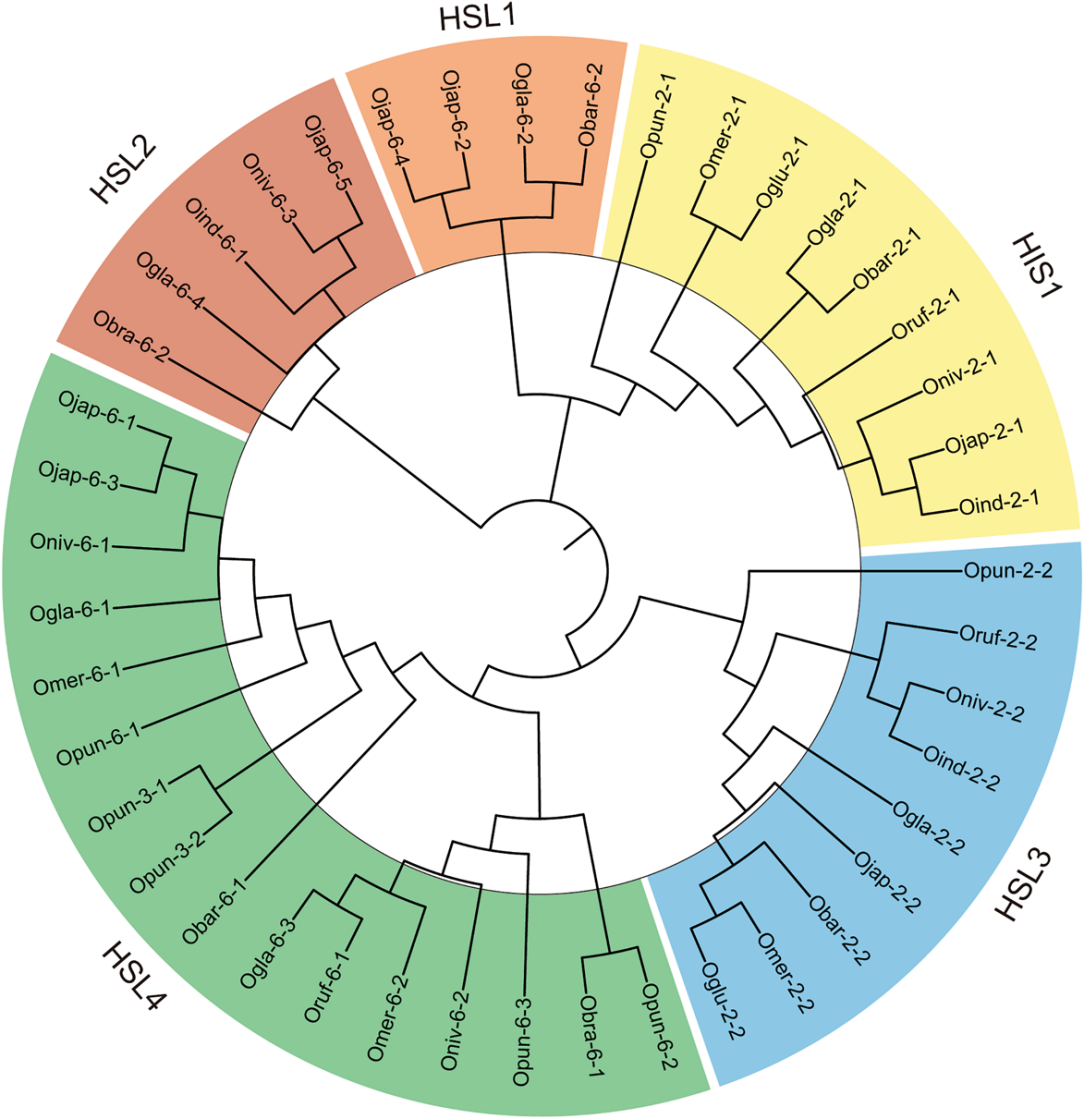
Phylogenetic tree of the *HIS1* and *HSL* gene families. The phylogenetic neighbor-joining tree was constructed using the amino acid sequences of *HIS1* and *HSL* orthologs in 10 *Oryza* species. In the *HSL* family, four subclasses (*HSL1*, *HSL2*, *HSL3*, and *HSL4*) were identified. Gene classes are indicated by the label colors, where 9 *HIS1* were labeled in yellow, 4 *HSL1* in orange, 5 *HSL2* in red, 9 *HSL3* in blue, and 16 *HSL4* in green.

**Figure 4:**
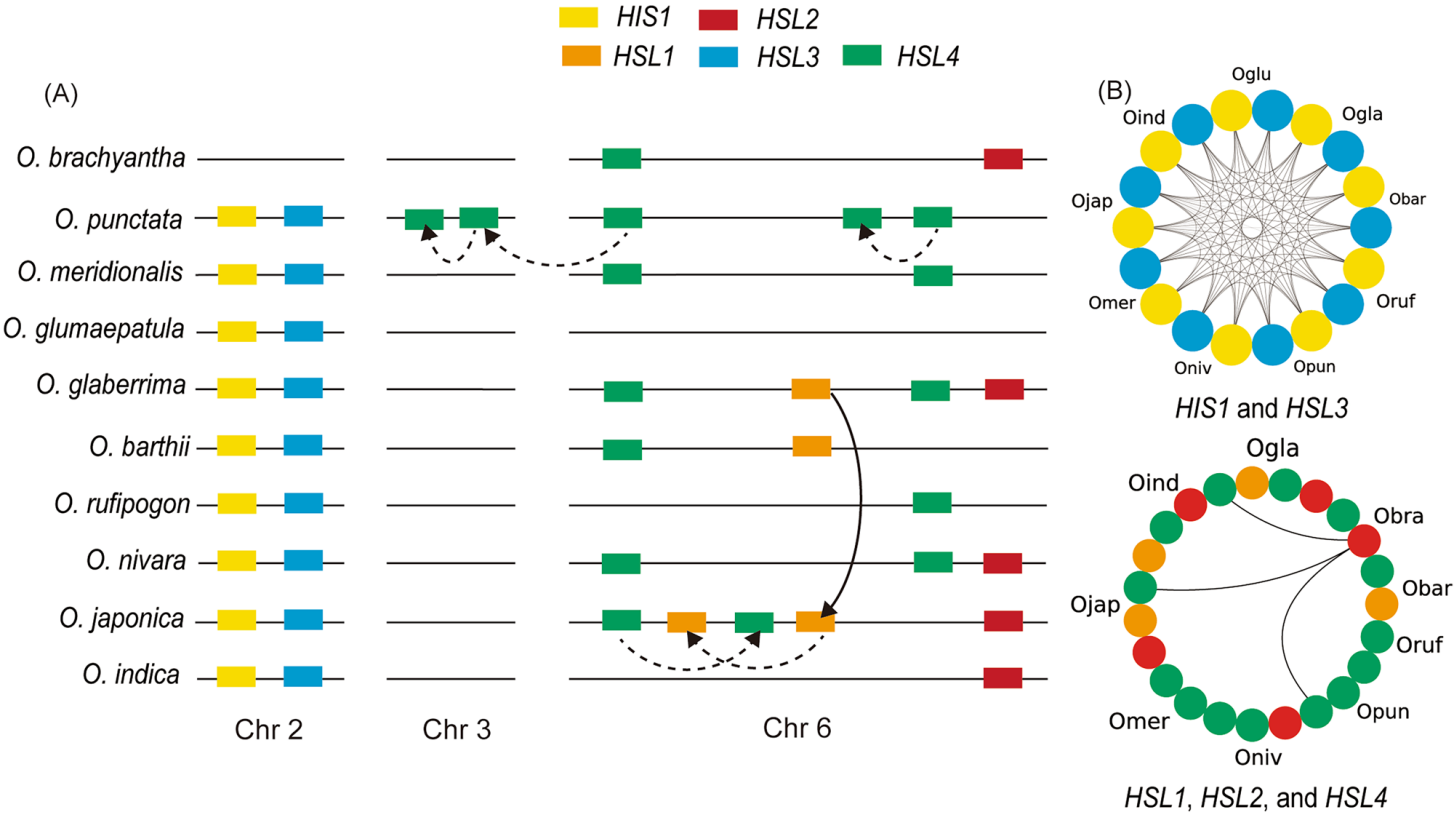
Chromosomal distribution and syntenic network of *HIS1* and *HSL* gene families. (**A**) The location of *HIS1* and *HSL* gene families at rice chromosomes 2, 3, and 6. Rectangles represented genes and their colors indicate the gene classes. The dotted and solid lines indicate the gene duplication and transfer events, respectively. (**B**) Syntenic network of *HIS1* and *HSL3* (upper) and *HSL1*, *HSL2*, and *HSL4* (lower). The lines represent the syntenic connection between the two genes and the circle color indicates the subclass of the gene family.

The phylogenetic tree was constructed using 43 *HIS1* orthologous genes and was further clustered into five subclasses consisting of *HIS1* and *HSL 1–4* based on the annotation data of *O. sativa *ssp.* japonica* (Fig. 3). In each clade, 9, 4, 5, 9, and 16 genes were grouped as *HIS1*, *HSL1*, *HSL2*, *HSL3*, and *HLS4*, respectively (Fig. 3). *HIS1* and *HSL* genes are tandemly located on chromosomes 2, 3, and 6 (Fig. 4A). *HIS1* and *HSL3* genes are located on chromosome 2 in all *Oryza* species except for *O. brachyantha* (Fig. 4A). *HSL1*, *HSL2*, and *HSL4* are mostly located on chromosome 6 in *Oryza* species and additional two *HSL4* are located in chromosome 3 in *O. punctata* (Fig. 4A). In *O. sativa *ssp.* japonica*, one copy of *HSL1* and *HSL4* were identified in chromosome 6, and the *HSL1* gene was only detected in *O. glaberrima*, *O. barthii*, and *O. sativa *ssp.* japonica* (Fig. 4A). The synteny analysis showed that all *HIS1* and *HSL3* orthologs had syntenic relationships in a pair-wise manner, while the synteny of *HSL1*, *HSL2*, and *HSL4* were not maintained among most of the *Oryza* species (Fig. 4B).

### Gene synteny analysis of *GBSS1*, *GBSS2*, and *BADH2*

**Figure 5:**
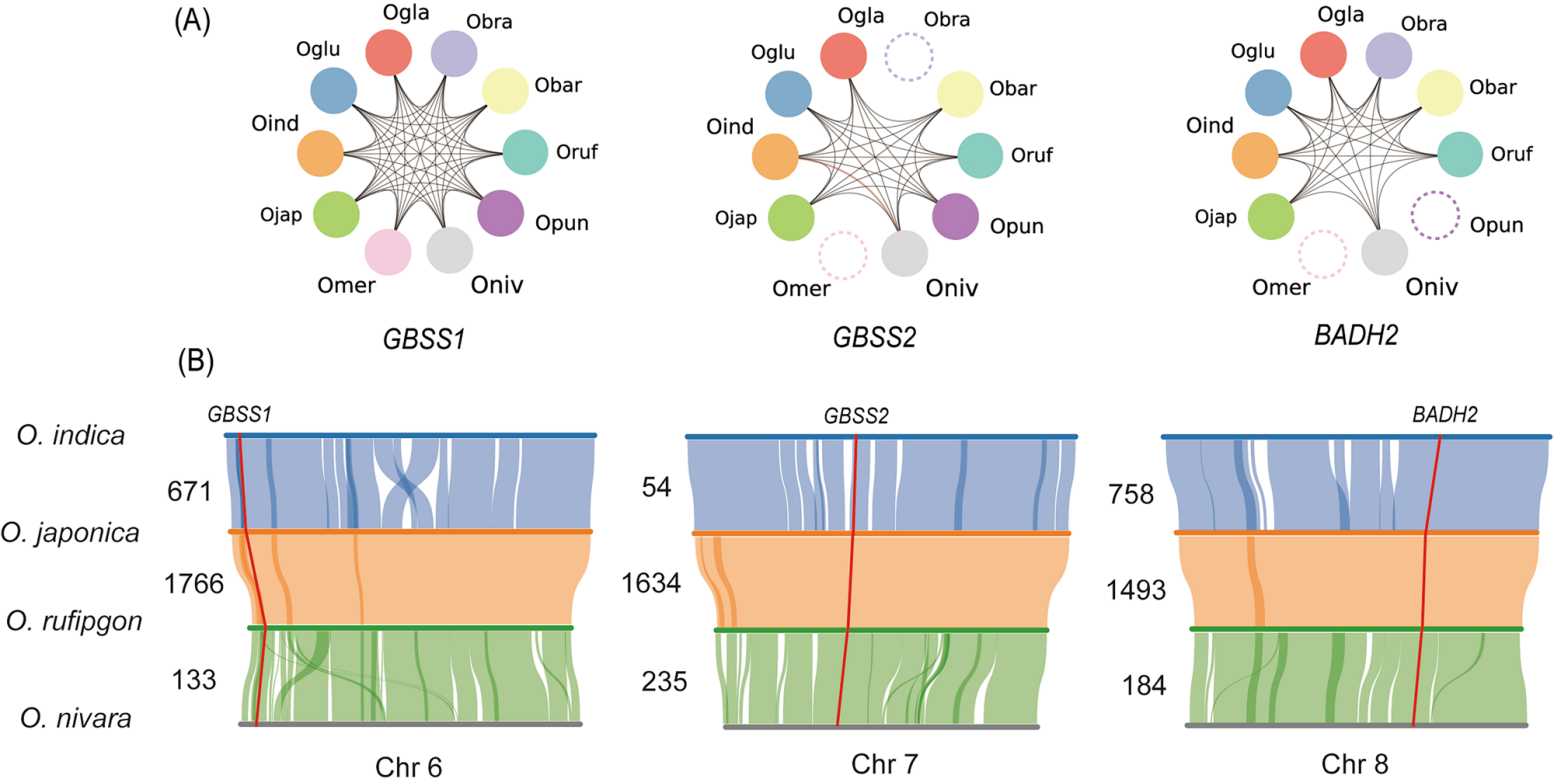
Syntenic conservation of *GBSS1*, *GBSS2*, and *BADH2*. (**A**) Syntenic network of the three genes across the *Oryza* species. The circles represent the orthologous genes detected by sequence similarity, dashed circles indicate the absence of the gene, and the lines represent a syntenic connection between the two genes. The line colors indicate whether the two genes are connected, and gray or red indicates the presence or absence thereof. (**B**) The feature of synteny blocks harboring the three genes among the Asian species including *O. sativa *ssp.* indica*, *O. sativa *ssp.* japonica*, *O. rufipogon*, and *O. nivara*. The red lines show the location and syntenic linkage of the orthologs. The numbers beside each synteny represent the number of genes in the synteny block of the focal genes.

Among the four Asian species, synteny analysis was conducted for *GBSS1*, *GBSS2*, and *BADH2*, which had a low copy number variation (Table 1). Among all the *Oryza* species with orthologous genes, including orthologs that were identified based on their sequence similarity, the synteny was highly conserved between the target genes (Fig. 5A, Table S1). The synteny blocks harboring *GBSS1*, *GBSS2*, and *BADH2*, were identified on chromosomes 6, 7, and 8, respectively (Fig. 5B). In the synteny blocks of *GBSS1*, there are 671 genes when comparing *O. sativa* ssp. *indica* with *O. sativa *ssp.* japonica*, 1766 genes when comparing *O. sativa *ssp.* japonica* with *O. rufipogon*, and 133 genes when comparing *O. rufipogon* with *O. nivara* (Fig. 5B). The *GBSS2* block contained 54, 1634, and 235 gene numbers. The block of *BADH2* had 758, 1493, and 184 gene numbers (Fig. 5B).

### Selection pressure

**Figure 6:**
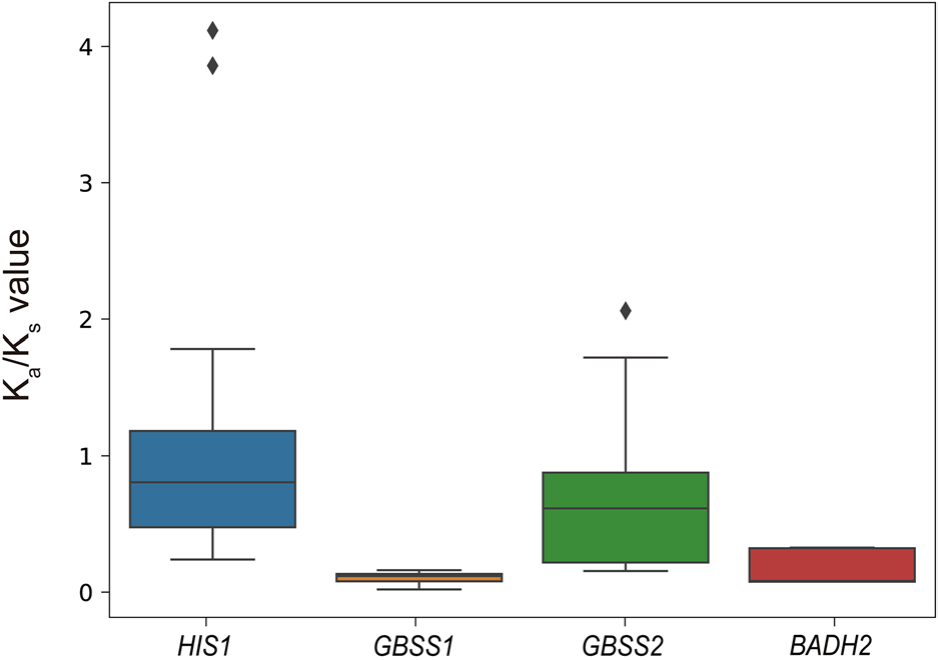
Comparison of the pair-wise *K*_a_/*K*_s_ values of *HIS1*, *GBSS1*, *GBSS2*, and *BADH2* genes. The *K*_a_/*K*_s_ values of the ortholog pairs were calculated in each of the focal genes to determine the selection pressure. The ortholog genes were obtained based on the synteny block among the 10 *Oryza* species.

The *K*_a_/*K*_s_ ratio of the homologs was calculated to determine whether *BADH2*, *GBSS1*, *GBSS2*, and *HIS1* underwent negative or positive selection (Fig. 6). The mean *K*_a_/*K*_s_ values for *HIS1*, *GBSS1*, *GBSS2*, and *BADH2* were 0.92, 0.1, 0.69, and 0.16, respectively, indicating that these genes evolved under purifying selection (Fig. 6). Moreover, the mean *K*_a_/*K*_s_ value of the *GBSS1* and *BADH2* gene pair were lower than those of the means other two families, which suggests that the *GBSS1* and *BADH2* duplicates evolved at a slower rate (Fig. 6). Additionally, we calculated the *K*_a_/*K*_s_ values by comparing target orthologs from two monocot plants, *Sorghum bicolor* (*S. bicolor*) and *Lessia perrie* (*L. perrie*), with those in *Oryza* species. The comparisons between *S. bicolor* and *Oryza* species showed *K*_a_/*K*_s_ values of 0.2, 0.13, and 0.14 for *GBSS1*, *GBSS2*, and *BADH2*, respectively (Table S2). When comparing *L. perrie* with *Oryza* species, the *K*_a_/*K*_s_ values were 0.12 and 0.11 for *GBSS1* and *BADH2*, respectively (Table S2). These results indicate that the target genes had been undergone purifying selection within cereal plants.

## Discussion

The evolution and speciation of Asian rice species have remained unclear due to their high ortholog sequence similarity^1,24^. To date, there are two hypotheses concerning the origin and domestication of the *O. sativa* species^24^. The single-domestication hypothesis posits that the *O. sativa* species originated from a wild ancestor and the differentiation between *O. sativa *ssp.* indica* and *O. sativa *ssp.* japonica* occurred after domestication of the cultivated species^25–28^. This single-domestication hypothesis is mainly supported by the molecular evidence of the identical sequences of the key domestication genes between the *O. sativa* subspecies, including *sh4* that reduces shattering and *prog1* which is associated with erect growth^25–27^. In contrast, the multiple-domestication hypothesis postulates that *O. sativa *ssp.* indica* and *O. sativa *ssp.* japonica* were domesticated separately from different wild ancestors^29,30^. This multiple-domestication hypothesis has gained support through the phylogenetic analyses which shows that the *O. sativa* subspecies are separated into distinct clades and are closer to the different wild accessions than each other^29,30^. The phylogenetic tree that was constructed in this study using the true orthologs based on syntenic relationship was consistent with the single-domestication hypothesis as the *O. sativa* subspecies were grouped into the same clade (Fig. 1). The synteny analysis revealed that the genomic structure of the *O. sativa* species was more conserved with *O. rufipogon* than those of *O. nivara* (Fig. 2B). This result is in agreement with a previous study which reported that the *O. sativa* species may originate from *O. rufipogon* and that *O. nivara* is one of the ecological varieties of *O. rufipogon*^25–27^.

Genomic similarity is a key factor that determines genetic compatibility which enables the transfer of desired traits between two species through interspecific crossing^31^. In rice, several reproductive isolations had been observed in interspecific hybrids, which resulted in inviability, weakness, and sterility^32^. Several genetic models have been suggested to explain the mechanisms of reproductive isolations in plants and structural variations were identified as one of leading factors of reproductive isolation^32^. In rice and Arabidopsis, pollen incompatibility and inviability had been reported in their hybrids, respectively, due to a change of the gene locus caused by reciprocal gene loss of duplicated genes^20,33^. Other studies have reported that chromosomal rearrangements enhance the reproductive isolation by suppressing recombination^31,34,35^, which results in unbalanced gametes that may be inviable^31,34^.

Suppressed recombination also increased the extent of linkage disequilibrium (LD) block, thereby restricting gene flow in potentially larger genomic regions^34,36,37^. When introgression of a favorable gene from an external germplasm into a cultivar occurs, other genes that confer undesirable traits can also be transferred if the genes are located in the same LD block^38,39^. This phenomenon is also known as the linkage drag problem and it is a major concern in introgression breeding which prevents breeders from introducing desirable traits into elite cultivars^40–42^. Linkage drag that leads to the negative relationships among the yield potential, grain quality, and environmental resistance have been reported in *O. sativa* species^40,41^. The synteny analysis in this study identified that *O. rufipogon* would be a more genetically compatible germplasm for *O. sativa* breeding with reduced reproductive isolation and linkage drag problems (Fig. 2).

Gene evolution analysis has been widely used to investigate gene expansion, domestication process, genetic background, etc. Copy number variation (CNV) is a structural variation that alters the dosage of genes, which could result in phenotypic changes^43,44^. In plants, most resistance traits are polygenic and highly affected by CNV^43,44^. A previous study on durum wheat reported that frost resistance levels were determined by the CNV of the *CBF-A14* gene family^45^. In rice, the CNV of 28 functional genes was identified to be involved in insect resistance and response to salt stress^43^. In *Brassica napus*, 563 resistance genes experienced 1137 CNV events including 704 deletions and 433 duplications^46^. Based on the phylogenetic clustering, a total of 43 *HIS1* genes were further clustered into five subclasses including *HIS1* (9), *HSL1* (4), *HSL2* (5), *HSL3* (9), and *HLS4* (16) (Fig. 3). We identified that the *HIS1* and *HSL* families experienced multiple duplication events (Fig. 4). In *O. punctata*, a tandemly duplicated *HSL4* gene was identified on chromosome 6 and an additional pair of tandemly duplicated *HSL4* genes were detected on chromosome 3 (Fig. 4). Because these *HSL4* genes of *O. punctata* were not found in other *Oryza* species, they might be duplicated after the *O. punctata* speciation event (Fig. 4). The *HSL1* genes were only identified in *O. glaberrima*, *O. barthii*, and *O. sativa *ssp.* japonica* (Fig. 4). Considering that *O. glaberrima* was domesticated from *O. barthii*, the *HSL1* gene probably originated from *O. barthii*, and then moved to *O. satvia *ssp.* japonica* via *O. glaberrima* or directly from *O. barthii* (Fig. 4). In *O. sativa *ssp.* japonica*, duplication events of *HSL1* and *HSL4* were identified on chromosome 6 (Fig. 4). Because four of the five duplicated *HSL1* and *HSL4* genes are located close to each other (less than 50 kb interval), we propose that the *HIS1* and *HSL* families were mainly expanded through tandem duplication events in the *Oryza* species (Fig. 4). These results are in agreement with a previous study where tandem duplication events were frequently identified in CNVs^47^. Our gene evolution analysis can facilitate the improvement in herbicide resistance of rice cultivars through gene transferring from wild germplasm that has a high copy number of *HIS1* and *HSL* genes such as *O. punctata* (Fig. 4).

While *HIS1* has diverse CNVs across 10 *Oryza* species, *GBSS1*, *GBSS2*, and *BADH2* genes have at most one copy in the 10 *Oryza* species and the syntenic relationship of their orthologs was deeply conserved in pair-wise comparison among *Oryza* species (Table 1 and Fig. 5A). These results suggest that these three genes descended from a common *Oryza* ancestor to present-day cultivars (Fig. 5). The *BADH2* and *GBSS2* loss events were identified in *O. brachyantha*, *O. punctata*, and *O. meridionalis*, which suggests that the functional role of the *BADH2* and *GBSS2* genes were developed after speciation from *O. meridionalis* (Table 1). Meanwhile, no loss event of *GBSS1* was identified in the *Oryza* species (Table 1). Plants have some critical genes that play essential roles in their survival such as photosynthesis, cell division, and reproduction^48–50^. In general, the genetic diversity of essential genes is highly conserved among related species, because the malfunction of these genes directly affects their fitness in the population^48^. In rice, a previous study reported that *GBSS1* is expressed in the endosperm and pollen grains, while the expression of *GBSS2* is limited to the vegetative tissues and pericarp^7^. The starch content of the endosperm serves as the primary source for seed germination and seedling growth^7^. Therefore, the prevalence of *GBSS1* may be the product of selection pressure for its critical role in seed germination vigor, as wild relatives and landraces with lower germination rates were extinguished in nature or removed from the breeding pool during rice evolution and domestication. This result is consistent with our selection pressure analysis which showed that *GBSS1* had the lowest *K*_a_/*K*_s_ ratio indicating that the sequence diversity of *GBSS1* is highly conserved during evolution (Fig. 6).

**Figure 7:**
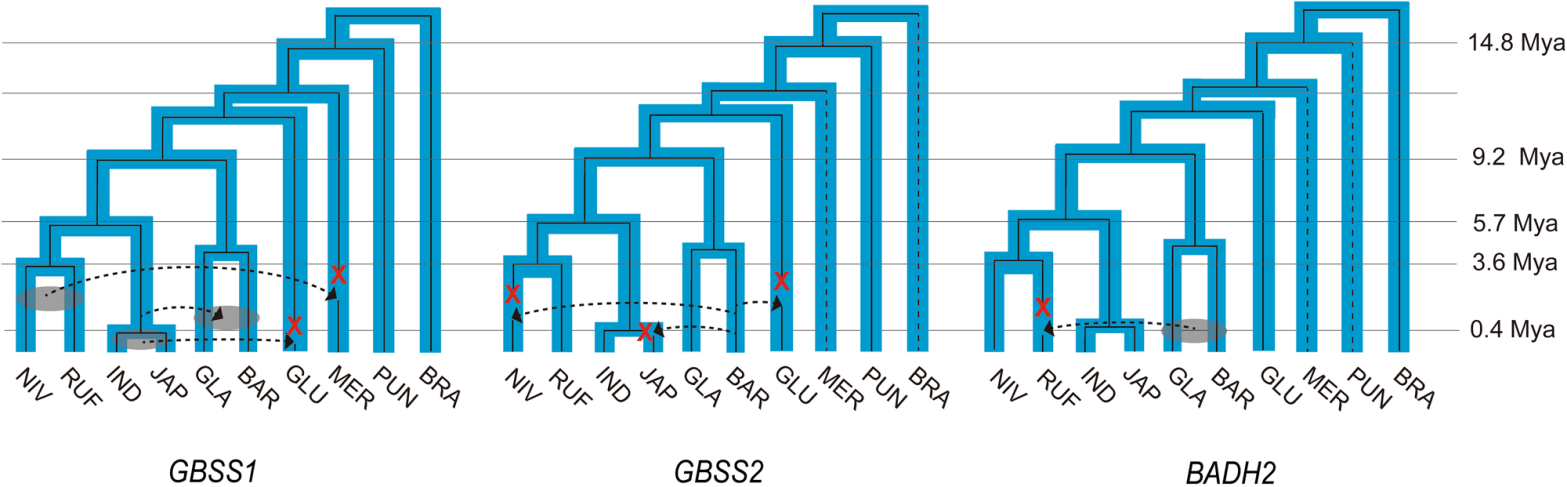
Schematic illustration of phylogenetic inference to detect gene transfer events. The species tree is depicted by the thick blue area and the inner black lines represent the phylogeny of each gene. The dotted line indicates the absence of the gene in the node. The red x symbol indicates the loss of the gene in the node. The translucent oval between two species represent that either a species or another species contributes to transfer event. The arrows indicate the gene transfer between two species or node.

Gene exchange is a key evolutionary mechanism that enhances the adaptability of the population against environment stresses^51^. Natural introgression between the Asian cultivated and wild species is common because they are often sympatric^24,51,52^. The African cultivated species have a relatively limited gene pool in the wild species compared to Asian rice and several historic introgression events from the Asian species are reported^24^. *GBSS1*, *GBSS2*, and *BADH2* genes are located in highly conserved synteny blocks as single-copy genes over the 10 *Orzya* species, which indicates they are true orthologs in the genus *Orzya*. Using the true orthologs of *GBSS1*, *GBSS2*, and *BADH2*, reconciled gene trees were constructed and estimated divergence time was calculated between orthologous gene pairs, to investigate gene transfer events of the target genes across the *Oryza* species (Fig. S1 and Table 2). Our results proposed that three transfer events had occurred in *GBSS1* from the Asian groups into the African group (*O. glaberrima* and *O. barthii*) and other wild species (*O. glumaepatula* and *O. meridionalis*) (Fig. 7). In contrast, the *GBSS2* was transferred from *O. barthii* into the Asian species including *O. sativa *ssp.* japonica* and *O. nivara* (Fig. 7). This gene flow in the opposite direction between Asian and African groups indicates that *GBSS1* and *GBSS2* gained their subfunctions independently in the Asian and African rice populations, respectively, and they were spread to other regions during the domestication process. For *BADH2*, a gene from either *O. barthii* or *O. glaberrima* was moved into *O. rufipogon*, which suggests that most *BADH2* alleles in *japonica* rice had been developed after divergence between the Asian and African groups (Fig. 7)^6,53^.

**Table 2:** The estimated divergence time for pairs of transferred orthologs.

Genes	Orthologous pair	*K*_a_	*K*_s_	Estimated divergence time (mya)
*GBSS1*	*O. nivara–O. meridionalis*	0.0015	0.0234	1.80
	*O. rufipogon–O. meridionalis*	0.0022	0.0234	1.80
	*O. sativa–O. barthi*	0.0007	0.0091	0.70
	*O. sativa–O. glaberrima*	0.0007	0.0091	0.70
	*O. sativa–O. glumaepatula*	0.0000	0.0045	0.35
*GBSS2*	*O. barthi–O. glumaepatula*	0.0014	0.0087	0.67
	*O. barthi–O. sativa *ssp.* japonica*	0.0058	0.0068	0.52
	*O. barthi–O. nivara*	0.0027	0.0065	0.50
*BADH2*	*O. barthi–O. rufipogon*	0.0000	0.0055	0.42

Overall, this study enhances our knowledge of the gene family evolution in rice and offers practical implications for rice breeding efforts, which ultimately supports the development of improved rice varieties with enhanced adaptability and productivity.

## Material and methods

### Data source

The peptide sequences, nucleotide sequences, and gene location information (GFF format) of three domesticated species (*O. satvia *ssp.* japonica*, *O. sativa *ssp.* indica*, and *O. glaberrima*), seven wild species (*O. rufipogon*, *O. nivara*, *O. barthii*, *O. brachyantha*, *O. glumaepatula*, *O. meridionalis*, and *O. punctata*), and two dicot plants (*A. thaliana* and *G. max*) were downloaded from EnsemblPlants (https://plants.ensembl.org, accessed on 17 April 2023) and Rice Genome Hub (https://rice-genome-hub.southgreen.fr, accessed on 17 April 2023) (Table S3). The protein and nucleotide sequences were filtered out and the longest isoform per gene was retained for downstream analysis.

### Species phylogenetic tree

Synteny analysis was conducted to obtain true orthologs across the 12 species (see section “Synteny detection” for detail). In our study, single-copy orthologous genes located in synteny blocks among 10 *Oryza* species were defined as true orthologous gene. A total of 50 syntenic orthologs, which are shared between *O. sativa *ssp.* japonica* and the 11 other species, were selected as true orthologs for phylogenetic analysis. Peptide and nucleotide sequences of the 50 true orthologs were aligned using ClustalOmega v1.2.4^54^ and then concatenated. Phylogenetic trees were built using RAxML v8.2.12 with 1000 bootstrap replicates^55^. Two maximum-likely hood models, JJT and GTRGAMMA, were used for phylogenetic tree of peptide and nucleotide sequences, respectively^55^. The phylogenetic tree was visualized using Interactive Tree Of Life^56^. The divergence time between the nodes was estimated by the MCMCtree package embedded in PAML v4.10.6^57^.

### Synteny detection

The macrosynteny blocks among the 12 species were identified using BLASTP version 2.9.0+ and software MCScanX^58,59^. The protein sequences of the homolog pairs were obtained by all-against-all BLASTP search with the standard parameter setting for MCScanX analysis (evalue: 1e−10, num alignments: 5, and outfmt: 6). The BLASTP outputs with the gene location data were imported into the MCScanX to identify the synteny blocks with the default parameters (match score: 50, gap penalty: − 1, match size: 5, evalue: 1e−5, and max gaps: 25)^58^. The synteny was visualized using SynVisio^56^.

### Identification of *BAHD2*, *GBSS1*, *GBSS2*, and *HIS1* orthologs

The protein sequences of the 12 species were clustered into orthologous groups using OrthoFinder v2.5.4 with sequence similarity searches conducted using DIAMOND^60^. Based on the gene annotation of *O. sativa *spp.* japonica*, we obtained the names of the four target genes: *BADH2* (Os08g0424500), *GBSS1* (Os06g0133000), *GBSS2* (Os07g0412100), and *HIS1* (Os02g0280700). The four orthologous group containing the focal genes of *O. satvia *ssp. *japonica* were selected as orthologs corresponding to each gene. To classify the *HIS1* orthologs into subclasses, their protein sequences were aligned and used to construct a neighbor-joining phylogenetic tree using MEGAX software with 1000 bootstrap replications^61^.

### Gene evolutionary analysis

The “add_ka_and_ks_to_collinearity.pl” script in MCScanX was used to determine the non-synonymous (*K*_a_) and synonymous (*K*_s_) substitution values of the homologs pairs^58^. The selection pressures were determined based on the *K*a/*K*s values of syntenic orthologs pairs in each focal orthologous group. The gene synteny of the focal orthologous groups was identified based on the macrosynteny analysis using an in-house developed python script. The functional annotation of genes in synteny block was conducted using grameen database (https://www.gramene.org/, accessed on 7 February 2024). To construct gene tree, protein sequences of true orthologs of *GBSS1*, *GBSS2*, and *BADH2* were aligned using ClustalOmega v1.2.4 with the default parameters^54^. Gene trees were constructed using RAxML v8.2.12 with 1000 bootstrap replicates^55^ and the trees were reconciled into the species tree to identify the transfer event using Notung v2.6.1.5 (Fig. S1)^62^. Divergence time (T) between orthologous pair was calculated based on a synonymous substitutions per year (λ) as T = *K*s/2 λ (λ = 6.5 × 10^−9^ for rice)^63^.

### Supplementary Information

Supplementary Information 1.

Supplementary Table S1.

## Data Availability

The datasets supporting the conclusions of this article are included within the article and its Supplementary Information.
