# Supplementary material for: Evolutionary and synteny analysis of HIS1, BADH2, GBSS1, and GBSS2 in rice: insights for effective introgression breeding strategies
Source: Sci Rep. 2024 Mar 4;14:5226. doi: 10.1038/s41598-024-55581-w (PMC10909864; doi:10.1038/s41598-024-55581-w)
Supplement: Supplementary file 1 — Supplementary Information 1. [file 41598_2024_55581_MOESM1_ESM.docx]

**Supplementary data**


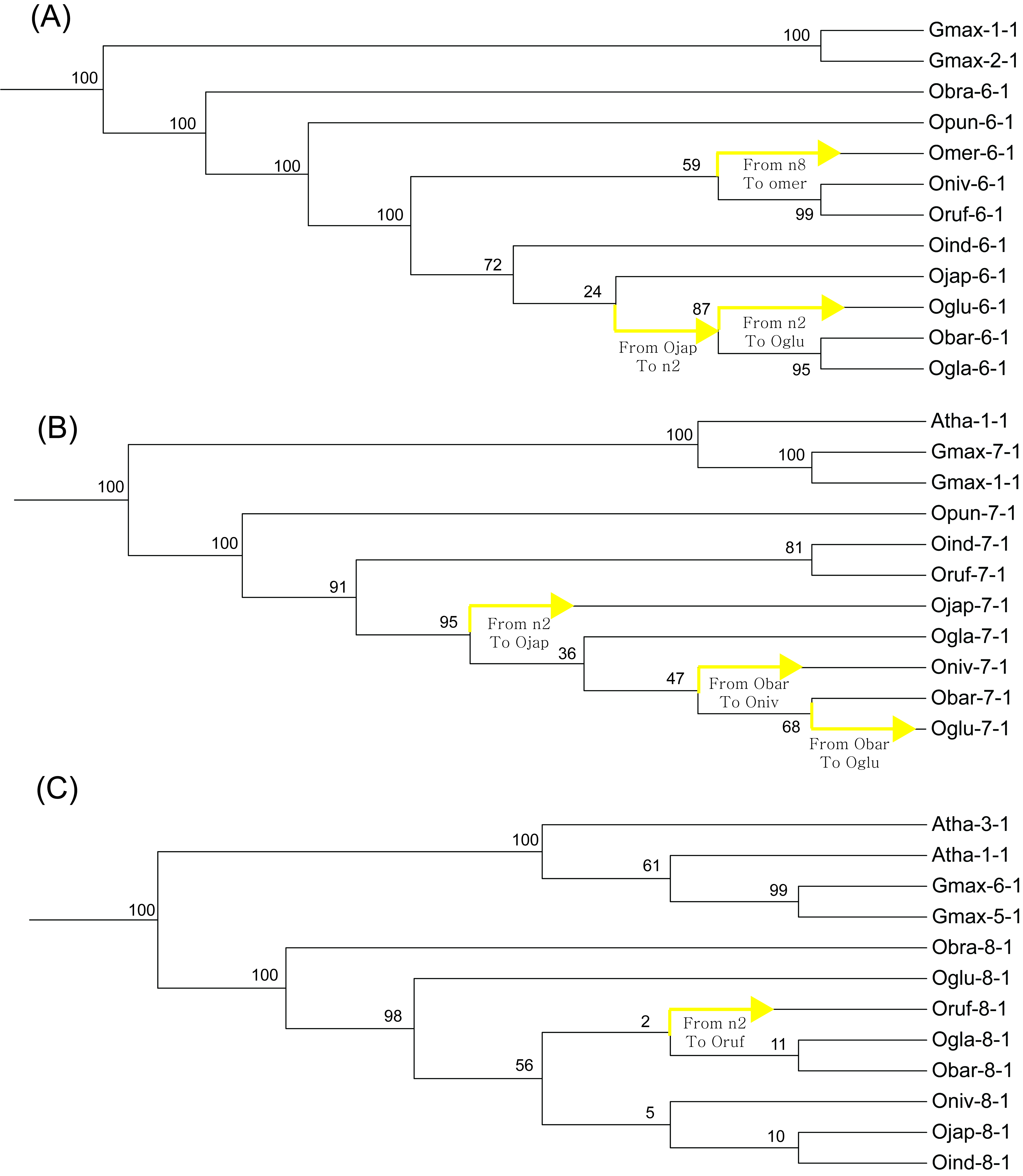


**Figure S1. Resolved gene tree of (A) *GBSS1*, (B) *GBSS2*, and (C) *BADH2***

The gene transfer events were investigated in 10 *Oryza* species. The yellow arrows indicate the transfer of genes from one node (n) or species to another species. The number of each node is the bootstrap value between nodes which is obtained from 1,000 bootstrap replications.


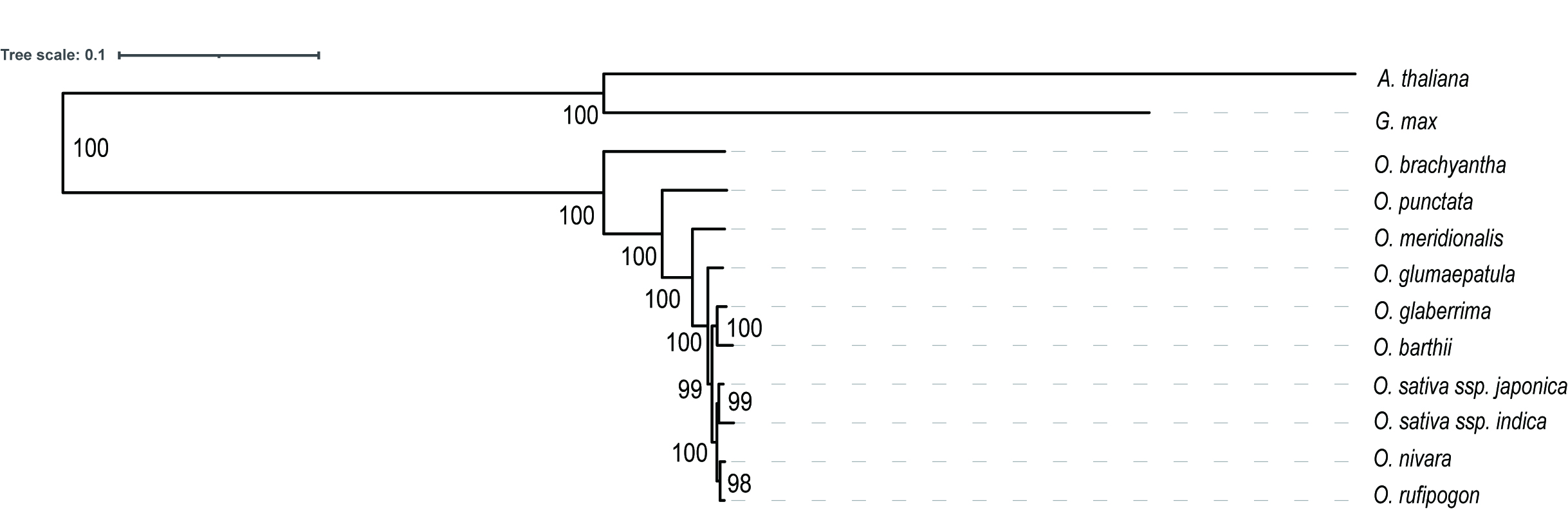


**Figure S2. Phylogenetic tree using nucleotide sequences.**

The phylogenetic tree was constructed using the nuecleotide sequences of 50 true orthologs among 10 *Oryza* species and two dicot species as outgroups. The number of each node is the bootstrap value between nodes which is obtained from 1,000 bootstrap replications.

**Supplementary Table 2. The estimated Ka/Ks values of orthologs.**

| Genes | Orthologous pair | *K*_a_ | *K*_s_ | *K*_a_/*K*_s_ | Estimated Divergence Time (Mya) |
| --- | --- | --- | --- | --- | --- |
| *GBSS1* | *S. bicolor-Oryza species* | 0.0925 | 0.4594 | 0.2 | 35.34 |
|  | *L. perrie-Oryza species* | 0.0551 | 0.3937 | 0.13 | 30.28 |
| *GBSS2* | *S. bicolor-Oryza species* | 0.0824 | 0.5549 | 0.14 | 42.69 |
| *BADH2* | *S. bicolor-Oryza species* | 0.0554 | 0.4285 | 0.12 | 32.96 |
|  | *L. perrie-Oryza species* | 0.0239 | 0.2157 | 0.11 | 16.59 |

**Supplementary Table 3. Genomic information of the 12 species used for this study**

| **Species**  **genome type (cultivar)** | **Origin** | **Type** | **Number of chromosomes** | **Number of genes** | **Download source** |
| --- | --- | --- | --- | --- | --- |
| *Arabiopsis thaliana* | Eurasia | cultivar | 5 | 27,497 | EnsemblPlants |
| *Glycine max* | Asia | cultivar | 20 | 55,589 | EnsemblPlants |
| *Oryza barthii* (AA) | Africa | wild | 12 | 34,575 | EnsemblPlants |
| *Oryza brachyantha* (FF) | Africa | wild | 12 | 32,037 | EnsemblPlants |
| *Oryza glaberrima* (AA) | Africa | cultivar | 12 | 30,018 | EnsemblPlants |
| *Oryza glumaepatula* (AA) | America | wild | 12 | 35,735 | EnsemblPlants |
| *Oryza meridionalis* (AA) | Australia | wild | 12 | 29,308 | EnsemblPlants |
| *Oryza nivara* (AA) | Asia | wild | 12 | 36,313 | EnsemblPlants |
| *Oryza punctata* (BB) | Africa | wild | 12 | 31,762 | EnsemblPlants |
| *Oryza rufipogon* (AA) | Asia | wild | 12 | 37,062 | EnsemblPlants |
| *Oryza sativa ssp. indica*  (AA; MH63) | Asia | cultivar | 12 | 60,122 | Rice Genome Hub |
| *Oryza sativa ssp. japonica*  (AA; Nipponbare) | Asia | cultivar | 12 | 35,427 | EnsemblPlants |
